# Lung Cancer Related Thrombosis (LCART): Focus on Immune Checkpoint Blockade

**DOI:** 10.3390/cancers16020450

**Published:** 2024-01-20

**Authors:** Andriani Charpidou, Grigorios Gerotziafas, Sanjay Popat, Antonio Araujo, Arnaud Scherpereel, Hans-Georg Kopp, Paolo Bironzo, Gilbert Massard, David Jiménez, Anna Falanga, Anastasios Kollias, Konstantinos Syrigos

**Affiliations:** 1 Third Department of Internal Medicine and Laboratory, Athens Medical School, National and Kapodistrian University of Athens, Sotiria General Hospital, 157 72 Athens, Greece; taskollias@gmail.com (A.K.);; 2 Assistance Publique-Hôpitaux de Paris, Thrombosis Center, Service D’Hématologie Biologique Hôpital Tenon, Sorbonne Université, 75005 Paris, France; 3 Royal Marsden Hospital, Institute of Cancer Research, London SW3 6JJ, UK; 4 Department of Medical Oncology, Centro Hospitalar Universitário do Porto, 4099-001 Porto, Portugal; antonio.araujo@chporto.min-saude.pt; 5 Department of Pulmonary and Thoracic Oncology, University of Lille, University Hospital (CHU), 59000 Lille, France; arnaud.scherpereel@chu-lille.fr; 6 Departments of Molecular Oncology and Thoracic Oncology, Robert-Bosch-Hospital Stuttgart, 70376 Stuttgart, Germany; 7 Department of Oncology, San Luigi Gonzaga Hospital, University of Turin, 10124 Turin, Italy; 8 Department of Thoracic Surgery, Hôpitaux Robert Schuman, 2540 Luxembourg, Luxembourg; 9 Respiratory Department, Ramón y Cajal Hospital, Instituto Ramón y Cajal de Investigación Sanitaria IRYCIS, 28034 Madrid, Spain; djimenez.hrc@gmail.com; 10 Department of Transfusion Medicine and Hematology, Hospital Papa Giovanni XXIII, University of Milan Bicocca, 24129 Bergamo, Italy

**Keywords:** anticoagulation, cancer-associated thrombosis, immune checkpoint inhibitor, lung cancer, venous thromboembolism

## Abstract

**Simple Summary:**

Thrombotic events are a common problem for lung cancer patients. Compared to other types of cancer, lung cancer patients are more likely to experience blood clots at any stage of their disease. Some newer cancer treatments, like immunotherapy, can also raise the risk of thrombosis. Two different categories of drugs that prevent the formation of blood clots, low-molecular-weight heparins (LMWHs) and direct oral anticoagulants (DOACs), have similar benefits in walking patients. The choice of which drug to use and when should be personalized based on the characteristics of the patient, the type of lung cancer, as well as recent treatments, such as surgery, chemotherapy or immunotherapy. The use of models that aim to predict the risk of blood clots for each patient may help physicians choose when to start treatment with anticoagulation drugs.

**Abstract:**

Cancer-associated thrombosis (CAT) is a common complication in lung cancer patients. Lung cancer confers an increased risk of thrombosis compared to other solid malignancies across all stages of the disease. Newer treatment agents, including checkpoint immunotherapy and targeted agents, may further increase the risk of CAT. Different risk-assessment models, such as the Khorana Risk Score, and newer approaches that incorporate genetic risk factors have been used in lung cancer patients to evaluate the risk of thrombosis. The management of CAT is based on the results of large prospective trials, which show similar benefits to low-molecular-weight heparins (LMWHs) and direct oral anticoagulants (DOACs) in ambulatory patients. The anticoagulation agent and duration of therapy should be personalized according to lung cancer stage and histology, the presence of driver mutations and use of antineoplastic therapy, including recent curative lung surgery, chemotherapy or immunotherapy. Treatment options should be evaluated in the context of the COVID-19 pandemic, which has been shown to impact the thrombotic risk in cancer patients. This review focuses on the epidemiology, pathophysiology, risk factors, novel predictive scores and management of CAT in patients with active lung cancer, with a focus on immune checkpoint inhibitors.

## 1. Introduction

Venous thromboembolism (VTE) represents a common but challenging disease entity in cancer patients. Approximately 10% of patients with cancer will develop cancer-associated thrombosis (CAT) each year [1]. Cancer represents a hypercoagulable and prothrombotic state, since irregularities may be observed in all elements of the Virchow’s triad: blood flow stasis, endothelial injury, and hypercoagulability [2].

The risk of VTE in cancer patients is 9 times higher than in the general population [3]. CAT is a major cause of death in cancer patients, as the mortality rate of people with cancer with CAT is 2- to 3-fold higher compared with those without CAT [4]. The risk of CAT is dependent on multiple factors, including cancer type, treatment modality, disease stage and time since diagnosis [1], with lung cancer patients being at a high risk of developing CAT. Up to one-fifth of lung cancer (LC) patients may be diagnosed with CAT during the natural history of the disease [5].

Different factors have been implicated in the development of CAT, including abnormalities in platelet count and function, increased expression of prothrombotic genes (particularly tissue factors), the circulation of tumor cells and cancer-associated microparticles, as well as consistent activation of the coagulation pathway [6,7]. Specifically in LC, increased levels of leukocytes, the generation of neutrophil extracellular traps (NETs), tissue factor-positive (TF+) microvesicles (MVs) and endothelial cell activation have been associated with CAT [8,9]. Furthermore, it was recently shown that LC patients demonstrate blood hypercoagulability characterized by decreased procoagulant phospholipid-dependent (Procoag-PPL) clotting time, the increased degradation of fibrin and exhausted platelets [9]. Interestingly, the ROADMAP-CAT study provided data suggesting that endothelial cell activation is among the dominant pathophysiological alterations in patients with lung adenocarcinoma [9].

In this review, we summarize advances in the management of CAT in LC patients including risk factors associated with thrombosis and bleeding, and the thrombotic risk attributed to antineoplastic agents used for the treatment of LC, with a focus on immune checkpoint inhibitors. Also, we discuss practical choices between the drugs available for long-term dynamic antithrombotic management, linked to their pharmacology, evidence of clinical benefits, and advantages and limitations in such a complex clinical context, to aid clinical decision-making that improves the care of patients with lung cancer.

## 2. Why Do Lung Cancer Patients Have a High Thrombotic Burden?

**Figure 1:**
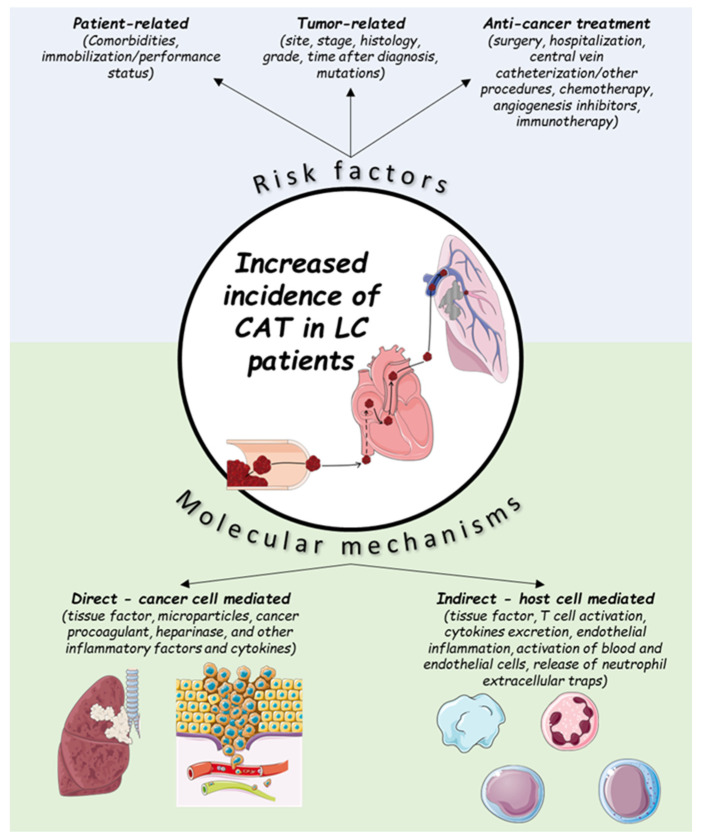
Risk factors and molecular mechanisms for cancer-associated thrombosis (CAT) in lung cancer (LC) patients.

Multiple factors account for the increased incidence of CAT in LC patients, and can be divided into two categories: individual patient- and cancer-associated factors [2,10]. Regarding the individual patient-related factors, the impact of age and sex is not clearly defined [2,11,12,13]. Co-morbidities such as anemia, obesity and chronic obstructive pulmonary disease (COPD) have been found to increase the VTE risk in LC patients; however, the effects of diabetes, hypertension, pulmonary tuberculosis and cardiovascular disease are not established [12]. Immobilization (can be evaluated clinically by the performance status) is also a significant risk factor, while the effect of smoking appears to be non-significant [2,12]. Regarding the cancer-related factors, these can be further divided into factors that are associated with the tumor itself or with the anticancer treatment [2,12,13] (Figure 1). LC is usually diagnosed in the advanced or metastatic disease stage [14], which is known to confer an increased risk of thrombosis [15]. The association of locally advanced or metastatic disease and VTE in LC patients has been demonstrated in large-cohort studies (locally advanced stage, adjusted HR: 2.9 (95% CI: 2.3–3.5), *p* < 0.001; metastatic stage, adjusted HR: 2.5 (95% CI:2.3–2.7), *p* < 0.001) [16]. VTE occurrence remains an important predictor for early mortality in metastatic NSCLC patients [17].

The role of circulating tumor cells (CTCs) in promoting CAT in LC patients is currently under investigation [18]. Computational models based on lung cancer patients have demonstrated the potential role of CTCs in reducing blood flow and the generation of thrombi in the lung vasculature [19]. These models were validated in a recent postmortem study, in which cancer cell clusters were detected in the majority of patients with pulmonary thrombosis [20].

Time since LC diagnosis is an important risk factor in CAT, as most thromboembolic events in patients with LC undergoing chemotherapy tend to occur within 6 months of diagnosis [5], for a 6.8% overall incidence of thromboembolic events and a median time to VTE event of 49 days [21]. Almost half of the VTE events occur in the first 3 months, and in approximately one-third of LC patients, VTE occurs within the first month of diagnosis [16].

Mucin-producing adenocarcinomas represent the histology most commonly associated with VTE in LC [22], demonstrating a three-fold higher risk (incidence: 66.7/1000 person-years) than patients with squamous cell carcinoma of the lung (incidence: 21.2/1000 person-years). A large-cohort study comprising 10,598 individuals with LC after a long follow-up of 14 years revealed that the adenocarcinoma subtype was an independent risk factor of VTE [23]. Increased microvesicular tissue factor activity has been detected in patients with adenocarcinoma, and this may be implicated in the higher rate of thrombosis for this histologic type [24]. Tumor grade may also play a role in the development of thrombosis, as patients with high-grade tumors are approximately twice as likely to develop CAT within 6 months [25]. A recent prospective trial of lung cancer patients demonstrated a trend towards a higher risk of VTE in patients with higher-grade tumors, although this association was not statistically significant after multivariate analysis [16].

The interaction between driver mutations and the risk of CAT is complex. The California Cancer Registry [26] identified an increased thromboembolic risk in ALK+ and ROS1+ NSCLC. In a recent meta-analysis and systematic review of eight studies, the pooled OR was 2.10 (95% CI: 1.70–2.60) for ALK+ NSCLC and 3.15 (95% CI: 1.83–5.43) for ROS1+ NSCLC [27]. A similar association has been shown in LC patients with KRAS mutations in a small case–control study [28]. In contrast, EGFR mutations have not been shown to increase the risk of CAT and may have a protective role [29]. The presence of driver mutations with anti-thrombotic effects may account for the lower risk of CAT in never-smokers with lung adenocarcinoma, a cohort associated with the presence of driver mutations, as has been demonstrated in some observational studies [16]. Larger prospective studies are required to further elucidate the effects of driver mutations on thrombotic risk in LC patients (Table 1).

Various molecular mechanisms have been suggested to address the high VTE incidence rate among LC patients [2,10] (Figure 1). These mechanisms can be either direct or indirect [2]. Direct mechanisms include the release of procoagulant factors from the tumor itself, which directly activate the thrombosis cascade [2,13]. These factors include: tissue factor, microparticles, cancer procoagulant, heparinase, and other inflammatory factors and cytokines [2,10,13,30]. Indirect mechanisms include the interaction and activation of host cells (endothelial or inflammation cells), which can also activate the coagulation cascade through various mechanisms (e.g., release of procoagulants, such as tissue factor) [2,10]. In the case of immunotherapy, additional immune-related mechanisms may be present, similar to those responsible for the other ICI-related adverse events (e.g., colitis, pneumonitis, etc.) [31].

## 3. Thrombotic Risk Is Related to Antineoplastic Treatment

Cancer patients undergoing systemic treatment for their malignancy are among the highest-risk populations for CAT. The effect of antineoplastic therapy on the overall incidence of CAT is complex, as while systemic treatment may increase the risk of thrombosis, the risk of CAT is reduced when tumor response is achieved [32]. Chemotherapy has been identified as an independent risk factor for CAT events in patients with LC [23,28,33,34]. The real-life incidence of CAT in patients with LC receiving chemotherapy has been reported as 14.1% at six months after the start of chemotherapy [35]. The risk of CAT is not consistent among all cytotoxic agents, including agents with the same mechanism of action [32]. In particular, platinum-based chemotherapy, which is widely used in LC patients, may increase the incidence of CAT. A retrospective analysis by the Memorial Sloan Kettering Cancer Center found that 18.1% of cancer patients developed thrombosis during cisplatin treatment within 4 weeks after chemotherapy [34]. Different mechanisms have been implicated in the development of chemotherapy-induced thrombosis, including direct endothelial damage and increased tissue factor activity [36]. The exact mechanisms of thrombosis for carboplatin and cisplatin, which are widely used in the treatment of LC, have yet to be determined [37].

LC patients with driver mutations receiving targeted therapies are also at a high risk of CAT [38]. However, as some driver mutations in LC have also been associated with an increase in risk (ALK, ROS1), it has not been established if the use of targeted agents has a beneficial or deleterious effect on the risk of CAT [29]. A recent retrospective cohort study suggested that the use of TKIs does not increase the risk of thrombosis, as the initiation of targeted therapy is not associated with an increased risk of CAT [39]. The mechanism of targeted therapy-induced thrombosis may be different depending on the type of agent used. The use of anti-vascular endothelial growth factor (VEGF) agents in advanced NSCLC has been associated with an increased risk of high-grade arterial thromboembolism, but not VTE [40]. The risk of CAT in patients receiving a combination treatment with targeted agents may be increased [41].

CAT represents a major contributor to morbidity and mortality in patients with early and locally advanced LC receiving treatment with curative intent [42]. Even in early stages, LC patients are in a hypercoagulable state characterized by the increased generation of thrombin and phosphatidylserine expressing platelet-derived microparticles (Pd-MP/PS+). This hypercoagulable state is not sufficiently corrected after lung lobectomy [43]. Locally advanced disease, requiring open and more extensive resections, has been associated with an increased risk of CAT, which doubles the risk of 1-year mortality in this population [42]. The use of radiotherapy has also been shown to increase the risk of CAT in LC patients [22].

Supportive treatments, including the use of red blood cell (RBC) transfusions, erythropoietin stimulating agents (ESAs) and central venous catheters (CVC), have been associated with CAT [44]. However, in a retrospective analysis of CAT trends in chemotherapy-induced anemia, the use of RBC transfusions or ESAs did not alter the risk of thrombosis in LC patients [45]. In contrast, CVC-related thrombosis (CRT) is a common complication in patients with indwelling central venous access devices. The widespread use of CVC in LC patients has altered the clinical presentation of CAT, including by increasing the rate of DVT in the upper extremities.

### Immune Checkpoint Blockade

The introduction of immune checkpoint inhibitors has altered the treatment landscape of LC, with demonstrably large benefits in overall survival but largely unknown effects on the risk of CAT. CAT is common in cancer patients receiving immunotherapy either as single-agent or combination regimens [46]. Lung cancer patients receiving immune checkpoint inhibitors may demonstrate a similar or higher risk of CAT compared to patients receiving chemotherapy, with the highest risk group being patients receiving combinations of chemotherapy and immunotherapy [38,47]. The real-life incidence of CAT in LC patients receiving therapy with immune checkpoint inhibitors is higher than 10% [48]. The use of combination immunotherapy with PD-1 and CTLA-4 blockade may increase the risk of CAT over PD-1 blockade alone (1-year incidence: 29.3% vs. 9.1–14.9%, *p* < 0.05) [49]. Pulmonary embolism is the cause of a significant proportion of CAT events in patients receiving immunotherapy [48]. CAT may be associated with higher mortality in this population, although this effect has not been consistently demonstrated across all studies [50].

The interplay between thrombosis and immune response, also described as immunothrombosis, has been well-established, most recently in patients with uncontrolled immune activation caused by COVID-19 [51]. In cancer patients, the formation of altered cell components during carcinogenesis that trigger the activation of innate immunity may promote immune-mediate thrombosis [52]. This process has been suggested to promote immune evasion and to inhibit the response to immune checkpoint blockade in cancer patients by altering T cell responses [53]. Furthermore, the development of fibrin clots alters the tumor immune microenvironment and may further inhibit immune response by presenting a physical barrier for infiltrating immune cells [54]. On the other hand, sustained inflammation has been associated with CAT and tumor metastasis in preclinical lung cancer models, including by the formation of neutrophil extracellular traps [55], which have been implicated in venous and arterial thrombosis as a response to inflammation [56]. Other suggested mechanisms of immunothrombosis in LC patients include the formation of platelet-T cell aggregates and the increased expression of tissue factors in circulating monocytes [20,57].

The exact mechanism for the development of CAT in LC patients receiving checkpoint immunotherapy has not been established. Factors supporting the inflammatory theory of thrombosis in patients receiving immune checkpoint inhibitors include the increased risk of thrombosis in patients receiving combination immunotherapy or experiencing immune-related adverse events, as well as correlations between CAT and inflammatory biomarkers such as C-reactive protein, interleukin-8 and myeloid-derived suppressor cells [49,58,59]. The PD-1/PD-L1 axis may also be directly involved, as PD-L1 positivity has been associated with an increased risk of VTE [60]. The potential mechanisms of immunothrombosis in LC are summarized in Table 2**.**

Therapeutic anticoagulation has not been shown to improve response rates or survival outcomes in patients receiving checkpoint inhibitors [61]. In a study of lung cancer patients receiving checkpoint immunotherapy, the use of anticoagulation at baseline demonstrated a trend towards worse survival, although this may be explained by the worse clinical status of patients that required anticoagulation [62]. Novel therapeutic strategies targeting immunothrombosis have been developed, including agents that suppress complement activation, which has been associated with CAT [53]. The combination of PD-1 blockade and AON-D21, an inhibitor of the complement factor C5a, has shown promise in preclinical lung cancer models [63]. However, this strategy is not yet ready to enter clinical practice. In STELLAR-001, a phase I trial of avdoralimab, a monoclonal antibody that inhibits the binding of C5a to the receptor C5aR1, in combination with durvalumab, a PD-L1 inhibitor, the combination therapy demonstrated limited clinical activity, with no responses observed in NSCLC patients that had previously received immunotherapy, and the trial was terminated early [64].

## 4. Risk Assessment Models (RAMs) for Cancer-Associated Thrombosis

The first RAM for CAT in outpatients with solid tumors, the Khorana Risk Score (KRS), was presented by Khorana et al. in 2008 [65]. The predictors of the KRS include the tumor type dichotomized to very high-risk cancers (stomach, pancreas) and high-risk cancers (lung, lymphoma, gynecologic, bladder, testicular, renal). The KRS also includes the body mass index as a patient-related factor. Lastly, the KRS includes hematological markers (pre-chemotherapy levels of hemoglobin, platelets and white blood cell count) that are non-specific for blood hypercoagulability. The original KRS has been derived and validated to evaluate VTE risk in outpatients before the initiation of chemotherapy [66]. The KRS may also be useful in hospitalized cancer patients. In a study by Patell et al., patients with a high KRS (≥3) were significantly more likely to develop VTE during hospitalization than patients with a low KRS (multivariable OR, 2.52; 95% CI, 1.31 to 4.86). Similar results were reported in a multicenter retrospective study of 1398 hospitalized patients [67,68]. In this analysis, in-hospital VTE occurred in 5.4% (95% CI, 1.9% to 8.9%) of high-risk patients, 3.2% (95% CI, 2.0% to 4.4%) of intermediate-risk patients, and 1.4% (95% CI, 0.3% to 2.6%) of low-risk patients (OR for high- low-risk patients, 3.9; 95% CI, 1.4 to 11.2).

However, the KRS has several limitations as a predictive and prognostic tool in clinical practice. In a recent multicenter prospective observational study in Japan, the KRS was evaluated in 1008 patients with advanced lung cancer, of whom 10% developed CAT. VTE risk could not be determined because both the Khorana score and modified Khorana score, based on BMI targets adjusted for the Asian population, showed very low areas under the curve (0.518 and 0.516, respectively) [69]. This result was replicated in a large systematic review and meta-analysis of 55 cohorts, where the KRS was of limited use for predicting future risk of CAT in LC patients [66]. Furthermore, the CASSINI study, a double-blind, randomized trial involving 1080 high-risk ambulatory patients with cancer (KRS of ≥2, on a scale from 0 to 6) who received either rivaroxaban or placebo for up to 6 months, did not demonstrate any significant decrease in 6-month symptomatic VTE rate in the treatment group (6%) versus the control group (8.8%; hazard ratio, 0.66; 95% confidence interval (CI), 0.40 to 1.09; *p*  =  0.10) [70]. In contrast, AVERT, a double-blind trial of apixaban designed similarly to CASSINI, showed a significantly reduced 6-month rate of VTE (4.2%) in the apixaban group as compared to the placebo group (10.2%; hazard ratio, 0.41; 95%; CI = 0.26 to 0.65; *p* < 0.001) [71].

The aforementioned limitations of the KRS have led to the development of other RAMs, derived from prospective observational studies, although none of them have been externally validated in cancer patients as extensively as KRS. The clinical, biochemical and genetic criteria used for the assessment of thrombotic risk in the different RAMs are summarized in Table 3.

## 5. Evaluation of the Bleeding Risk

Bleeding is a significant challenge for patients with advanced solid tumors, with approximately 10% of all cancer patients having at least one episode [72]. Hemoptysis is among the most common respiratory symptoms in LC, and approximately 20–60% of patients with LC will experience some degree of hemoptysis during the natural history of the disease [73,74,75]. In total, 5–10% of episodes of hemoptysis are considered severe (blood loss > 100 mL/day), and without timely management, the mortality rate exceeds 50%.

Chemotherapy-induced thrombocytopenia (CIT) (defined as platelet count < 100 × 10^9^/L) can delay antineoplastic treatments or surgical procedures, while increasing the likelihood of serious bleeding events eventually resulting in hospitalization [76]. Importantly, while thrombopenia increases the risk of bleeding, it does not reduce the risk of CAT [77], complicating the use of anticoagulation, as most RCTs that evaluate its benefits in CAT exclude this patient population [78,79,80,81]. In addition, LC patients may develop organ insufficiencies either as a result of their malignancy or from adverse effects of different treatment modalities, including chemotherapy, immunotherapy and targeted therapy. In particular, renal and hepatic insufficiency increase the risk of both CAT and bleeding, while simultaneously affecting the pharmacokinetics of different anticoagulation agents [82,83,84]. Particularly for LC, squamous histology, vascular invasion, central location, and history of hemoptysis are related to an increased risk of bleeding.

A careful and comprehensive evaluation of the patient with active LC and an individualized approach tailored to the patient’s bleeding risk are key to the assessment and management of anticoagulation for CAT. In particular, clinicians should assess:

- The particular characteristics of LC in the evaluated patient, including lung cancer histology, the stage and resectability of the disease, the invasion of large vessels, the presence of active brain metastases, the response to antineoplastic treatment and the presence of cancer cachexia;
- The medications of the patient, including present antineoplastic and anticoagulation therapy and possible drug–drug interactions;
- The personal history of bleeding or thrombosis;
- The existence of comorbidities that exacerbate the risk of bleeding or CAT, including thrombopenia, renal or hepatic insufficiency, gastrointestinal and other disorders, as well as the expected duration and potential reversibility;
- The prognosis of the disease, the intent of treatment (curative or palliative) and patient preferences

Based on this individualized approach, clinicians should consider the appropriate anticoagulation strategy for each patient. In most cases, it is reasonable to offer low-molecular-weight heparin (LMWH), adjusted based on the anti-Xa activity level according to the risk of thrombus progression. Severe thrombocytopenia (platelet count < 50 × 10^9^/L) is not considered an absolute contraindication to full-dose anticoagulation in combination with appropriate supportive care strategies that include platelet transfusions and bleeding management. In general, anticoagulation should only be discontinued when the risk of bleeding is very high (e.g., active bleeding, platelet count < 25 × 10^9^/L, acute hepatitis, uncontrolled hypertension, acute stroke, etc.) [85].

## 6. Considerations when Choosing the Optimal Anticoagulant

At this point in time, the benefit of anticoagulation in CAT is well established. Large meta-analyses of RCTs of ambulatory patients that included LC patients have shown that both LMWHs and direct-acting oral anticoagulants (DOACs) confer an approximate 50% reduction in the risk of CAT without increasing the risk of major bleeding [86,87], and therefore are reasonable options. In LC patients at higher risk of bleeding (squamous histology, vascular invasion, central location, history of hemoptysis), it is reasonable to prefer LMWHs over DOACs due to the potentially lower risk of bleeding, based on indirect evidence [78]. Regardless of the specific agent chosen, patients at high risk of CAT should receive prophylactic anticoagulation for at least 6 months, with treatment beyond 6 months being a reasonable option for individual patients at very high risk of CAT [88]. In patients hospitalized for an acute medical illness, LMWHs should be preferred, as DOACs are associated with an increased risk of bleeding in this setting [88]. Vitamin K antagonists are less effective in reducing the risk of CAT and should be considered a second-line option for prophylaxis in patients ineligible for treatment with LMWHs or DOACs [89]. As the benefits and risks of different anticoagulation agents are similar in many cases, the selection of the optimal treatment strategy for the management of CAT should be individualized, based on the risk of thrombosis and bleeding, the potential for drug–drug interactions (DDIs), polypharmacy and patient preferences.

Polypharmacy, defined as the use of five or more medications, is common in patients with cancer, who are often treated with multiple antineoplastic and supportive therapies. The risk of major DDIs in cancer patients increases linearly from 14% in patients receiving less than four medications to 67% in patients receiving more than 11 medications [90]. Furthermore, the use of two or more medications has been associated with an increased risk of major bleeding in patients receiving anticoagulation for VTE or atrial fibrillation [91]. It is thus important to evaluate the potential for drug–drug interactions when selecting the appropriate anticoagulation therapy for CAT. All DOACs are substrates of P-glycoprotein and cytochrome P450, so therapies that affect P-glycoprotein or CYP3A4 metabolism have the potential to interact with DOACs [92]. Numerous anticancer therapies are inhibitors or inducers of the P-glycoprotein and/or CYP3A4 pathways, with the potential to interact with DOACs [93]. LC therapies with the potential for DDIs with DOACs, along with their bleeding, gastrointestinal and hematological implications, are depicted in Supplementary Table S1. Despite fewer interactions with DOACs, physicians need to also consider the pharmacokinetic DDIs of supportive care drugs and comorbidities when prescribing DOACs. On the other hand, there is little risk of pharmacokinetic DDIs with LMWHs [83]. The potential DDIs of supportive oncology care drugs are depicted in Table 4.

Given the potential for DDIs with DOACs, clinicians should consider the use of LMWHs in cases of polypharmacy, where appropriate. In all cases with a moderate to high risk of DDI, it is recommended that patients with CAT be referred for a pharmacist-led drug interaction evaluation, which should be repeated if lung cancer management changes.

In most cases where alterations in anticoagulant pharmacokinetics are likely (e.g., impaired renal function, obesity), LMWHs may be preferred over DOACs due to the lack of safety data for DOACs in this population, as well as the ability to titrate the dose of LMWHs based on anti-Xa activity [85]. Patient preferences should also be considered when choosing the optimal anticoagulation agent, as most patients are not able to adhere to LMWHs for a 3- or 6-month duration of treatment, in contrast to DOACs [95].

## 7. Diagnosis of Lung Cancer-Associated Thrombosis

Incidental CAT (ICAT) refers to DVT or PE that is clinically unsuspected at the time of the diagnosis [96]. Although IPE may be asymptomatic, nearly 2/3 of the affected patients report symptoms consistent with PE, such as fatigue or shortness of breath [97,98]. These non-specific symptoms are often attributed to cancer or to side effects of treatment. The discovery of incidental PE (IPE) tends to be radiologist (observer)-dependent [99]. Several studies that reassessed routine CT scans of cancer patients for IPE have reported false-negative rates ranging from 30% to 75% [100,101,102,103]. Up to half of all CAT events diagnosed in oncology centers are incidental [104,105,106,107,108]. ICAT has been associated with significantly reduced median overall survival and a high risk of recurrent CAT even when receiving anticoagulant treatment [99,106,109,110]. International clinical practice guidelines recommend standard anticoagulation, similar to symptomatic CAT [85,111,112].

## 8. Prophylaxis in the Surgical Setting

The incidence of CAT after LC surgery may be higher than previously suspected. A systematic review of 19 studies including data from 10660 patients undergoing surgery for LC found that the incidence of CAT events may be up to 19% (mean 2%) [113]. Most of the studies did not provide data regarding thromboprophylaxis strategy. Only five studies reported the bleeding rate, which varied from 0.6 to 4.5% [113]. In another systematic review including 22 trials and 9072 patients, the overall mean risk of CAT was estimated at 3.8% [114]. Finally, the incidence of postoperative CAT after thoracic surgery in general has been estimated at between 0.4% and 51% for DVT and from <1% to 5% for PE, with 2% of PE cases being fatal. Thoracic surgery patients must, therefore, be considered at high risk of postoperative CAT [115] and should be offered pharmacologic thromboprophylaxis [85,115,116]. Prophylaxis should be commenced preoperatively [115]. Extended prophylaxis with LMWH for up to 4 weeks postoperatively is recommended for high-thrombotic-risk patients [112].

## 9. Prophylaxis in the Medical Setting

Hospitalized medical cancer patients are at high risk of developing CAT, a major risk factor for in-hospital mortality [117]. Nearly half of real-world patients receiving chemotherapy for metastatic NSCLC are hospitalized during their therapy [118]. Out of 570,304 LC hospitalizations, 20,672 (3.6%) had a clinically relevant diagnosis of CAT resulting in significantly longer length of stay, higher mortality, increased costs and increased risk for moderate to severe disability upon discharge [119]. Hospitalized patients with active LC and acute medical illness should be offered thromboprophylaxis. Thromboprophylaxis should not be offered to patients admitted for the sole purpose of minor procedures, chemotherapy infusion or stem-cell/ bone marrow transplantation [112].

In ambulatory cancer patients, there is wide variability in the risk of symptomatic CAT, based on different cancer types, cancer stages, and anti-cancer and supportive treatments, as well as individual patient characteristics. Large studies with unselected cancer patients undergoing anti-cancer treatment showed significant reductions in CAT by prophylactic anticoagulation, but event rates and the absolute differences were low [70,71,120,121,122,123,124]. DOACs may be preferable to LMWH, as a result of their lower cost and easier route of administration [124,125]. High-risk outpatients with active LC (Khorana score ≥2 before starting a new systemic anticancer regimen) may be offered thromboprophylaxis with apixaban, rivaroxaban, or LMWH [85,112].

## 10. Treatment of Lung Cancer-Associated Thrombosis

The treatment of CAT comes with a heightened risk of anticoagulant-related bleeding that differs by choice of anticoagulant as well as by patient- and disease-specific risk factors. LMWH has demonstrated improved outcomes compared with VKAs [126,127,128,129,130,131,132]. In RCTs comparing VKAs with DOACs, cancer patients were under-represented [133,134,135,136,137,138]. A meta-analysis pooling results from cancer patients in these trials [133,134,135,136,137,138] found a non-significant reduction in the risk of recurrent CAT and major bleeding with DOACs vs. VKAs [139]. A meta-analysis of six RCTs comparing the efficacy and safety of DOACs vs. LMWHs [78,79,80,81,140,141] showed that DOACs are associated with a reduced risk of recurrent CAT, without a significant difference in major bleeding [142]. While DOACs have been associated with higher bleeding rates in gastrointestinal intraluminal tumors as well as tumors of the genitourinary tract, this association has not been demonstrated in LC [143]. DOACs or LMWHs should be used for most patients with CAT; VKA should be used only if DOACs or LMWHs are inappropriate or unavailable [16,17,18,56,57,58]. LMWHs may be preferred in patients with GI or GU intraluminal (particularly unresected) tumors. At least six months of therapy is recommended [89,112,144,145]. Switching to an alternate anticoagulant regimen or increasing the dose of LMWH is recommended for recurrent CAT [89,112,144].

## 11. Special Populations

Evidence shows that some baseline characteristics and/or comorbidities might influence the risk of CAT or other CAT-related outcomes in patients with LC [85,146]. Older age is a controversial risk factor for CAT in LC patients, and further investigation is needed [13,17,26,147]. Major comorbidities, including chronic obstructive pulmonary disease (COPD), chronic kidney disease and cardiovascular disease, as well as smoking and obesity, may also increase the risk of CAT [16,38,148,149,150,151,152,153]. In a recent meta-analysis of 16 studies, the prevalence of PE was significantly higher among LC patients with COPD history (OR =2.59; 95% CI, 1.09–6.15; *p* = 0.03) [149]. In a retrospective study including 632 patients with newly diagnosed LC, multivariable analysis found that hypertension was significantly associated with CAT in different tumor histologies and stages [151]. In a study including 950 LC patients, obesity significantly increased the risk for CAT (OR 2.40; 95% CI, 1.26–4.58) [150]. Concurrent atrial fibrillation has also been associated with CAT [154,155,156]. An individualized approach is key for the optimal management of anticoagulation in patients with active cancer [157].

## 12. Lung Cancer, CAT and COVID-19

Patients with LC are vulnerable to coronavirus disease 2019 (COVID-19) with a high risk of severe morbidity and mortality [158]. In January 2022, the National Lung Cancer Audit reported on the negative impact of COVID-19 on LC diagnosis and treatment pathways within the NHS [159]. Both LC and COVID-19 are well-established risk factors for CAT, with mechanisms involving the interaction of cancer cells or SARS-CoV-2 with the coagulation system and the endothelial cells [160,161,162]. The incidences of CAT and arterial thromboembolic events (ATE) in the COVID-19 and Cancer Consortium registry cohort study were 7.6% and 3.9%, respectively [163]. In a study using national data from France (*n* = 89,530 patients with COVID-19), patients with COVID-19 and cancer (*n* = 6201) had a higher risk of complications compared to patients without cancer [163]. On the other hand, some studies did not show a higher rate of thrombotic events in cancer patients with COVID-19 compared to non-cancer patients [164,165], findings that can be attributed to the heterogeneity in the diagnostic protocols across the studies.

Thromboprophylaxis for all hospitalized cancer patients with COVID-19 is recommended [162,166,167,168]. Higher (therapeutic) doses of thromboprophylaxis are recommended for high-thrombotic-risk, non-critically ill patients [169]; yet, this recommendation has not been specifically investigated in cancer patients. The COVID-TE risk assessment model could be useful to guide personalized clinical decisions [170]. Post-discharge patients with high CAT risk could receive thromboprophylaxis according to periodical re-evaluations [162,169]. Individualized thromboprophylaxis could be suggested in cancer outpatients with COVID-19 with a high CAT/disease worsening risk [162,169]. The treatment for established CAT is similar to that for patients without COVID-19.

## 13. Conclusions

Cancer-associated thrombosis represents a major risk factor for morbidity and mortality in lung cancer patients. The risk of CAT depends on various factors, related to patients, tumor characteristics and type of antineoplastic or supportive treatment.

New therapeutic approaches with direct oral anticoagulants (DOACs) for CAT have been under evaluation in recent years, trying to offer an alternative to the “all comers” use of low-molecular-weight heparins (LMWH). The use of DOACs for the prevention or treatment of thrombotic events in ambulatory lung cancer patients should be individualized by balancing the risk of bleeding and thrombosis in specific populations based on squamous histology, recent surgery, vascular invasion, central location and history of hemoptysis.

The broad use of checkpoint inhibitors in combination with chemotherapy or novel agents has altered the therapeutic landscape of metastatic, and recently also of operable, LC. Thus, the need for rigorous research into their impact on hypercoagulation or the bleeding risk of patients has become conspicuous. In addition, we should evaluate the effect of COVID-19 infection as a separate risk factor. Physicians will face the challenge of improving the risk assessment models for CAT by focusing on tumor type, and incorporating biomarkers including genetic or microRNA profiles.

## Figures and Tables

**Table 1:** The role of driver mutations in NSCLC in the development of CAT, adapted from [29].

Molecular Alteration	CAT Incidence	Effect on CAT Risk
ALK rearrangement	26.9–47.1%	2.2–5 times increase
ROS1 rearrangement	34.6–41.6%	3–5 times increase
KRAS mutation	16.1–54%	2.67 times increase
EGFR mutation	9–35%	Conflicting results

**Table 2:** Proposed mechanisms of immunothrombosis in LC patients.

Interaction of Thrombosis with Immune Response	Interaction of Immune Response with Thrombosis
Immune invasion	Neutrophil extracellular traps
Diminished T-cell response	Platelet T-cell aggregates
Altered tumor immune microenvironment	Tissue factor-positive monocytes

**Table 3:** Risk assessment models for cancer-associated thrombosis. Score criteria are provided where appropriate. Abbreviations: BC—breast cancer; CAT—cancer-associated thrombosis; ChT—chemotherapy; Hb—hemoglobin; KRS—Khorana risk score; VTE—venous thromboembolism; WBC—white blood cell. Cardiovascular risk is defined as at least 2 of the following: peripheral artery disease, ischemic stroke, coronary artery disease, hypertension, hyperlipidemia, diabetes, obesity.

Criteria	KRS	Vienna-CATS	CONKO	ONCOTEV	PROTECHT	COMPASS-CAT
Very high-risk tumor (stomach, pancreas)	2	2	2		2	
High-risk tumor (lung, lymphoma, gynecologic, bladder, testicular)	1	1	1		1	
Pre-ChT platelet count ≥ 350 × 10^9^/L	1	1	1		1	2
Hb ≤ 100 g/L or use of red cell growth factors	1	1	1		1	
Pre-ChT WBC ≤ 11 × 10^9^/L	1	1	1		1	
BMI ≥ 35 kg/m^2^ or more	1	1	1		1	
D-dimer > 1.44 μg/L		1				
Soluble P-Selectin > 53.1 ng/L		1				
WHO performance status ≥ 2			1			
Gemcitabine ChT					1	
Platinum-based ChT					1	
KRS > 2				1		
Previous VTE				1		1
Metastatic disease				1		
Vascular/lymphatic macroscopic compression				1		
Anti-hormonal therapy for BC or anthracycline ChT						6
Time since cancer diagnosis ≤6 months						4
Central venous catheter						3
Advanced disease						2
Cardiovascular risk						5
Recent hospitalization for acute medical illness						5
Low	0	0	0	0	0	0–6
Intermediate	1–2	1–2	1–2	1	1–2	
High	≥3	≥3	≥3	≥2	≥3	≥7

**Table 4:** Potential interactions of supportive oncology care agents [93,94]. Abbreviations: ESA, erythropoiesis-stimulating agent; G-CSF, granulocyte colony-stimulating factor; P-gp, P-glycoprotein.

Category	Agent	CYP3A4 Interactions	P-gp Interactions
Corticosteroids	Dexamethasone	Strong inducer and substrate	No
	Prednisolone	Moderate inducer and substrate	Inhibitor and Substrate
Bisphosphonates and Denosumab	Zoledronic acid	No	No
	Denosumab	No	No
Antiemetics	Ondansetron	Substrate	Substrate
	Palonosetron	Substrate	No
	Metoclopramide	No	No
	Aprepitant	Moderate inhibitor and substrate	No
	Fosaprepitant	Moderate inhibitor and substrate	No
Analgesics and anxiolytics	Oxycodone	Substrate	No
	Hydromorphone	No	No
	Morphine	No	No
	Fentanyl	Weak inhibitor and substrate	No
	Paracetamol	Weak inhibitor and substrate	No
	Lorazepam	No	No
	Clonazepam	Substrate	No
G-CSF	Filgrastim	No	No
ESA	Epoetin alfa/beta	No	No
	Darbepoetin alfa	No	No

## Supplementary Materials

The following supporting information can be downloaded at: https://www.mdpi.com/article/10.3390/cancers16020450/s1, Table S1: Lung cancer therapies with potential drug-drug interactions with direct oral anticoagulants (DOACs).

## References

[1] Horsted F., West J., Grainge M.J. (2012). Risk of Venous Thromboembolism in Patients with Cancer: A Systematic Review and Meta-Analysis. PLoS Med..

[2] Abdol Razak N., Jones G., Bhandari M., Berndt M., Metharom P. (2018). Cancer-Associated Thrombosis: An Overview of Mechanisms, Risk Factors, and Treatment. Cancers.

[3] Mulder F.I., Horváth-Puhó E., van Es N., van Laarhoven H.W.M., Pedersen L., Moik F., Ay C., Büller H.R., Sørensen H.T. (2021). Venous Thromboembolism in Cancer Patients: A Population-Based Cohort Study. Blood.

[4] Khorana A.A., Francis C.W., Culakova E., Kuderer N.M., Lyman G.H. (2007). Thromboembolism Is a Leading Cause of Death in Cancer Patients Receiving Outpatient Chemotherapy. J. Thromb. Haemost..

[5] Suzuki T., Fujino S., Inaba S., Yamamura R., Katoh H., Noji Y., Yamaguchi M., Aoyama T. (2020). Venous Thromboembolism in Patents with Lung Cancer. Clin. Appl. Thromb. Hemost..

[6] Noble S., Pasi J. (2010). Epidemiology and Pathophysiology of Cancer-Associated Thrombosis. Br. J. Cancer.

[7] Soff G.A. (2013). Pathophysiology and Management of Thrombosis in Cancer: 150 Years of Progress. J. Thromb. Thrombolysis.

[8] Hisada Y., Mackman N. (2017). Cancer-Associated Pathways and Biomarkers of Venous Thrombosis. Blood.

[9] Syrigos K., Grapsa D., Sangare R., Evmorfiadis I., Larsen A.K., Van Dreden P., Boura P., Charpidou A., Kotteas E., Sergentanis T.N. (2018). Prospective Assessment of Clinical Risk Factors and Biomarkers of Hypercoagulability for the Identification of Patients with Lung Adenocarcinoma at Risk for Cancer-Associated Thrombosis: The Observational ROADMAP-CAT Study. Oncologist.

[10] Nasser N.J., Fox J., Agbarya A. (2020). Potential Mechanisms of Cancer-Related Hypercoagulability. Cancers.

[11] Chew H.K., Wun T., Harvey D., Zhou H., White R.H. (2006). Incidence of Venous Thromboembolism and Its Effect on Survival Among Patients with Common Cancers. Arch. Intern. Med..

[12] Li Y., Shang Y., Wang W., Ning S., Chen H. (2018). Lung Cancer and Pulmonary Embolism: What Is the Relationship? A Review. J. Cancer.

[13] Di W., Xu H., Xue T., Ling C. (2021). Advances in the Prediction and Risk Assessment of Lung Cancer-Associated Venous Thromboembolism. CMAR.

[14] Walters S., Maringe C., Coleman M.P., Peake M.D., Butler J., Young N., Bergström S., Hanna L., Jakobsen E., Kölbeck K. (2013). Lung Cancer Survival and Stage at Diagnosis in Australia, Canada, Denmark, Norway, Sweden and the UK: A Population-Based Study, 2004–2007. Thorax.

[15] Fernandes C.J., Morinaga L.T.K., Alves J.L., Castro M.A., Calderaro D., Jardim C.V.P., Souza R. (2019). Cancer-Associated Thrombosis: The When, How and Why. Eur. Respir. Rev..

[16] Kuderer N.M., Poniewierski M.S., Culakova E., Lyman G.H., Khorana A.A., Pabinger I., Agnelli G., Liebman H.A., Vicaut E., Meyer G. (2018). Predictors of Venous Thromboembolism and Early Mortality in Lung Cancer: Results from a Global Prospective Study (CANTARISK). Oncologist.

[17] Su Y., Huo M., Hua L., Zhang Y., Yi J., Zhang S., Li J., Zhang Y. (2021). Association of Venous Thromboembolism and Early Mortality in Patients with Newly Diagnosed Metastatic Non-Small Cell Lung Cancer. Cancer Manag. Res..

[18] Tormoen G., Haley K., Levine R., McCarty O. (2012). Do Circulating Tumor Cells Play a Role in Coagulation and Thrombosis?. Front. Oncol..

[19] Phillips K.G., Lee A.M., Tormoen G.W., Rigg R.A., Kolatkar A., Luttgen M., Bethel K., Bazhenova L., Kuhn P., Newton P. (2015). The Thrombotic Potential of Circulating Tumor Microemboli: Computational Modeling of Circulating Tumor Cell-Induced Coagulation. Am. J. Physiol.-Cell Physiol..

[20] Gi T., Kuwahara A., Yamashita A., Matsuda S., Maekawa K., Moriguchi-Goto S., Sato Y., Asada Y. (2023). Histopathological Features of Cancer-Associated Venous Thromboembolism: Presence of Intrathrombus Cancer Cells and Prothrombotic Factors. Arterioscler. Thromb. Vasc. Biol..

[21] Madison C.J., Melson R.A., Conlin M.J., Gundle K.R., Thompson R.F., Calverley D.C. (2021). Thromboembolic Risk in Patients with Lung Cancer Receiving Systemic Therapy. Br. J. Haematol..

[22] Blom J.W., Osanto S., Rosendaal F.R. (2004). The Risk of a Venous Thrombotic Event in Lung Cancer Patients: Higher Risk for Adenocarcinoma than Squamous Cell Carcinoma. J. Thromb. Haemost..

[23] Walker A.J., Baldwin D.R., Card T.R., Powell H.A., Hubbard R.B., Grainge M.J. (2016). Risk of Venous Thromboembolism in People with Lung Cancer: A Cohort Study Using Linked UK Healthcare Data. Br. J. Cancer.

[24] Hisada Y., Thålin C., Lundström S., Wallén H., Mackman N. (2018). Comparison of Microvesicle Tissue Factor Activity in Non-Cancer Severely Ill Patients and Cancer Patients. Thromb. Res..

[25] Ahlbrecht J., Dickmann B., Ay C., Dunkler D., Thaler J., Schmidinger M., Quehenberger P., Haitel A., Zielinski C., Pabinger I. (2012). Tumor Grade Is Associated with Venous Thromboembolism in Patients with Cancer: Results from the Vienna Cancer and Thrombosis Study. J. Clin. Oncol..

[26] Chew H.K., Davies A.M., Wun T., Harvey D., Zhou H., White R.H. (2008). The Incidence of Venous Thromboembolism among Patients with Primary Lung Cancer. J. Thromb. Haemost..

[27] Zhu V.W., Zhao J.J., Gao Y., Syn N.L., Zhang S.S., Ou S.-H.I., Bauer K.A., Nagasaka M. (2021). Thromboembolism in ALK+ and ROS1+ NSCLC Patients: A Systematic Review and Meta-Analysis. Lung Cancer.

[28] Corrales-Rodriguez L., Soulières D., Weng X., Tehfe M., Florescu M., Blais N. (2014). Mutations in NSCLC and Their Link with Lung Cancer-Associated Thrombosis: A Case-Control Study. Thromb. Res..

[29] Leiva O., Connors J.M., Al-Samkari H. (2020). Impact of Tumor Genomic Mutations on Thrombotic Risk in Cancer Patients. Cancers.

[30] Vitale C., D’Amato M., Calabrò P., Stanziola A.A., Mormile M., Molino A. (2015). Venous Thromboembolism and Lung Cancer: A Review. Multidiscip. Respir. Med..

[31] Wang T.-F., Carrier M. (2023). Immune Checkpoint Inhibitors-Associated Thrombosis: Incidence, Risk Factors and Management. Curr. Oncol..

[32] Muñoz Martín A.J., Ramírez S.P., Morán L.O., Zamorano M.R., Benéitez M.C.V., Salcedo I.A., Escobar I.G., Fernández J.M.S. (2020). Pharmacological Cancer Treatment and Venous Thromboembolism Risk. Eur. Heart J. Suppl..

[33] Salla E., Dimakakos E.P., Tsagkouli S., Giozos I., Charpidou A., Kainis E., Syrigos K.N. (2016). Venous Thromboembolism in Patients Diagnosed with Lung Cancer. Angiology.

[34] Moore R.A., Adel N., Riedel E., Bhutani M., Feldman D.R., Tabbara N.E., Soff G., Parameswaran R., Hassoun H. (2011). High Incidence of Thromboembolic Events in Patients Treated with Cisplatin-Based Chemotherapy: A Large Retrospective Analysis. J. Clin. Oncol..

[35] Kenmotsu H., Notsu A., Mori K., Omori S., Tsushima T., Satake Y., Miki Y., Abe M., Ogiku M., Nakamura T. (2021). Cumulative Incidence of Venous Thromboembolism in Patients with Advanced Cancer in Prospective Observational Study. Cancer Med..

[36] Haddad T.C., Greeno E.W. (2006). Chemotherapy-Induced Thrombosis. Thromb. Res..

[37] Grover S.P., Hisada Y.M., Kasthuri R.S., Reeves B.N., Mackman N. (2021). Cancer Therapy–Associated Thrombosis. Arterioscler. Thromb. Vasc. Biol..

[38] Hill H., Robinson M., Lu L., Slaughter D., Amin A., Mileham K., Patel J.N. (2021). Venous Thromboembolism Incidence and Risk Factors in Non-Small Cell Lung Cancer Patients Receiving First-Line Systemic Therapy. Thromb. Res..

[39] Roopkumar J., Poudel S.K., Gervaso L., Reddy C.A., Velcheti V., Pennell N.A., McCrae K.R., Khorana A.A. (2021). Risk of Thromboembolism in Patients with ALK- and EGFR-Mutant Lung Cancer: A Cohort Study. J. Thromb. Haemost..

[40] Zhang D., Zhang X., Zhao C. (2016). Risk of Venous and Arterial Thromboembolic Events Associated with Anti-VEGF Agents in Advanced Non-Small-Cell Lung Cancer: A Meta-Analysis and Systematic Review. Onco Targets Ther..

[41] Girard N., Cho B.C., Spira A.I., Shu C.A., Sanborn R.E., Neal J.W., Marmarelis M.E., Sabari J.K., Waqar S.N., Nagasaka M. (2023). Risk Factors for Venous Thromboembolism (VTE) among Patients with EGFR-Mutated Advanced Non-Small Cell Lung Cancer (NSCLC) Receiving Amivantamab plus Lazertinib versus Either Agent Alone. JCO.

[42] Akhtar-Danesh G.-G., Akhtar-Danesh N., Shargall Y. (2022). Venous Thromboembolism in Surgical Lung Cancer Patients: A Provincial Population-Based Study. Ann. Thorac. Surg..

[43] Papageorgiou C., Vandreden P., Marret E., Bonnet F., Robert F., Spyropoulos A., Galea V., Elalamy I., Hatmi M., Gerotziafas G.T. (2013). Lobectomy and Postoperative Thromboprophylaxis with Enoxaparin Improve Blood Hypercoagulability in Patients with Localized Primary Lung Adenocarcinoma. Thromb. Res..

[44] Castaman G. (2016). Risk of Thrombosis in Cancer and the Role of Supportive Care (Transfusion, Catheters, and Growth Factors). Thromb. Res..

[45] Bryer E.J., Kallan M.J., Chiu T.-S., Scheuba K.M., Henry D.H. (2020). A Retrospective Analysis of Venous Thromboembolism Trends in Chemotherapy-Induced Anemia: Red Blood Cell Transfusion versus Erythrocyte Stimulating Agent Administration. EJHaem.

[46] Roopkumar J., Kim A.S., Bicky T., Hobbs B.P., Khorana A.A. (2018). Venous Thromboembolism in Cancer Patients Receiving Immunotherapy. Blood.

[47] Deschênes-Simard X., Richard C., Galland L., Blais F., Desilets A., Malo J., Cvetkovic L., Belkaid W., Elkrief A., Gagné A. (2021). Venous Thrombotic Events in Patients Treated with Immune Checkpoint Inhibitors for Non-Small Cell Lung Cancer: A Retrospective Multicentric Cohort Study. Thromb. Res..

[48] Bjørnhart B., Hansen K.H., Jørgensen T.L., Herrstedt J., Schytte T. (2021). Incidence, Risk Factors and Clinical Outcome of Venous Thromboembolism in Non-Small Cell Lung Cancer Patients Receiving Immune Checkpoint Inhibition. Thromb. Update.

[49] Le Sève J.D., Guédon A.F., Bordenave S., Agard C., Connault J., Pistorius M.-A., Quéreux G., Espitia O. (2023). Risk Factors of Venous Thromboembolic Disease in Cancer Patients Treated with Immune Checkpoint Inhibitor. Thromb. Haemost..

[50] Wang T.-F., Khorana A.A., Carrier M. (2021). Thrombotic Complications Associated with Immune Checkpoint Inhibitors. Cancers.

[51] Bonaventura A., Vecchié A., Dagna L., Martinod K., Dixon D.L., Van Tassell B.W., Dentali F., Montecucco F., Massberg S., Levi M. (2021). Endothelial Dysfunction and Immunothrombosis as Key Pathogenic Mechanisms in COVID-19. Nat. Rev. Immunol..

[52] Engelmann B., Massberg S. (2013). Thrombosis as an Intravascular Effector of Innate Immunity. Nat. Rev. Immunol..

[53] Bauer A.T., Gorzelanny C., Gebhardt C., Pantel K., Schneider S.W. (2022). Interplay between Coagulation and Inflammation in Cancer: Limitations and Therapeutic Opportunities. Cancer Treat. Rev..

[54] Wahab R., Hasan M.M., Azam Z., Grippo P.J., Al-Hilal T.A. (2023). The Role of Coagulome in the Tumor Immune Microenvironment. Adv. Drug Deliv. Rev..

[55] Wolach O., Martinod K. (2022). Casting a NET on Cancer: The Multiple Roles for Neutrophil Extracellular Traps in Cancer. Curr. Opin. Hematol..

[56] Thålin C., Hisada Y., Lundström S., Mackman N., Wallén H. (2019). Neutrophil Extracellular Traps. Arterioscler. Thromb. Vasc. Biol..

[57] Meikle C.K., Meisler A.J., Bird C.M., Jeffries J.A., Azeem N., Garg P., Crawford E.L., Kelly C.A., Gao T.Z., Wuescher L.M. (2020). Platelet-T Cell Aggregates in Lung Cancer Patients: Implications for Thrombosis. PLoS ONE.

[58] Moik F., Riedl J., Barth D., Chan W.-S.E., Wiedemann S., Höller C., Fuereder T., Jost P., Pabinger I., Preusser M. (2022). Early Dynamics of C-Reactive Protein Predict Risk of Venous Thromboembolism in Patients with Cancer Treated with Immune Checkpoint Inhibitors. Blood.

[59] Roopkumar J., Swaidani S., Kim A.S., Thapa B., Gervaso L., Hobbs B.P., Wei W., Alban T.J., Funchain P., Kundu S. (2021). Increased Incidence of Venous Thromboembolism with Cancer Immunotherapy. Medicne.

[60] Söyler Y., Akın Kabalak P., Kavurgacı S., Akyürek N., Demirağ F., Yılmaz Ü. (2023). Could PD-L1 Positivity Be Associated with Venous Thrombosis in Patients with Non-Small Cell Lung Cancer?. J. Thromb. Thrombolysis.

[61] Johannet P., Sawyers A., Gulati N., Donnelly D., Kozloff S., Qian Y., Floristan A., Hernando E., Zhong J., Osman I. (2021). Treatment with Therapeutic Anticoagulation Is Not Associated with Immunotherapy Response in Advanced Cancer Patients. J. Transl. Med..

[62] Nichetti F., Ligorio F., Zattarin E., Signorelli D., Prelaj A., Proto C., Galli G., Marra A., Apollonio G., Porcu L. (2020). Is There an Interplay between Immune Checkpoint Inhibitors, Thromboprophylactic Treatments and Thromboembolic Events? Mechanisms and Impact in Non-Small Cell Lung Cancer Patients. Cancers.

[63] Ajona D., Ortiz-Espinosa S., Moreno H., Lozano T., Pajares M.J., Agorreta J., Bértolo C., Lasarte J.J., Vicent S., Hoehlig K. (2017). A Combined PD-1/C5a Blockade Synergistically Protects against Lung Cancer Growth and Metastasis. Cancer Discov..

[64] Bennouna J., Touchefeu Y., Ghiringhelli F., Isambert N., Barlesi F., Tomasini P., Cassier P., Edeline J., Sourd S.M.L., Tosi D. (2022). 15P STELLAR-001: A Phase I Study of the Anti-C5aR Avdoralimab in Combination with the Anti-PD-L1 Durvalumab in Advanced Solid Tumors. Ann. Oncol..

[65] Khorana A.A., Kuderer N.M., Culakova E., Lyman G.H., Francis C.W. (2008). Development and Validation of a Predictive Model for Chemotherapy-Associated Thrombosis. Blood.

[66] Mulder F.I., Candeloro M., Kamphuisen P.W., Di Nisio M., Bossuyt P.M., Guman N., Smit K., Büller H.R., van Es N. (2019). CAT-prediction collaborators The Khorana Score for Prediction of Venous Thromboembolism in Cancer Patients: A Systematic Review and Meta-Analysis. Haematologica.

[67] Patell R., Rybicki L., McCrae K.R., Khorana A.A. (2017). Predicting Risk of Venous Thromboembolism in Hospitalized Cancer Patients: Utility of a Risk Assessment Tool. Am. J. Hematol..

[68] Parker A., Peterson E., Lee A.Y.Y., de Wit C., Carrier M., Polley G., Tien J., Wu C. (2018). Risk Stratification for the Development of Venous Thromboembolism in Hospitalized Patients with Cancer. J. Thromb. Haemost..

[69] Tsubata Y., Kawakado K., Hamai K., Furuya N., Yokoyama T., Saito R., Nakamura A., Masuda T., Hamaguchi M., Kuyama S. (2023). Identification of Risk Factors for Venous Thromboembolism and Validation of the Khorana Score in Patients with Advanced Lung Cancer: Based on the Multicenter, Prospective Rising-VTE/NEJ037 Study Data. Int. J. Clin. Oncol..

[70] Khorana A.A., Soff G.A., Kakkar A.K., Vadhan-Raj S., Riess H., Wun T., Streiff M.B., Garcia D.A., Liebman H.A., Belani C.P. (2019). Rivaroxaban for Thromboprophylaxis in High-Risk Ambulatory Patients with Cancer. N. Engl. J. Med..

[71] Carrier M., Abou-Nassar K., Mallick R., Tagalakis V., Shivakumar S., Schattner A., Kuruvilla P., Hill D., Spadafora S., Marquis K. (2019). Apixaban to Prevent Venous Thromboembolism in Patients with Cancer. N. Engl. J. Med..

[72] Johnstone C., Rich S.E. (2018). Bleeding in Cancer Patients and Its Treatment: A Review. Ann. Palliat. Med..

[73] Gershman E., Guthrie R., Swiatek K., Shojaee S. (2019). Management of Hemoptysis in Patients with Lung Cancer. Ann. Transl. Med..

[74] Hu P., Wang G., Cao H., Ma H., Sui P., Du J. (2013). Haemoptysis as a Prognostic Factor in Lung Adenocarcinoma after Curative Resection. Br. J. Cancer.

[75] Reck M., Barlesi F., Crinò L., Henschke C.I., Isla D., Stiebeler S., Spigel D.R. (2012). Predicting and Managing the Risk of Pulmonary Haemorrhage in Patients with NSCLC Treated with Bevacizumab: A Consensus Report from a Panel of Experts. Ann. Oncol..

[76] Weycker D., Hatfield M., Grossman A., Hanau A., Lonshteyn A., Sharma A., Chandler D. (2019). Risk and Consequences of Chemotherapy-Induced Thrombocytopenia in US Clinical Practice. BMC Cancer.

[77] Kopolovic I., Lee A.Y.Y., Wu C. (2015). Management and Outcomes of Cancer-Associated Venous Thromboembolism in Patients with Concomitant Thrombocytopenia: A Retrospective Cohort Study. Ann. Hematol..

[78] Raskob G.E., van Es N., Verhamme P., Carrier M., Di Nisio M., Garcia D., Grosso M.A., Kakkar A.K., Kovacs M.J., Mercuri M.F. (2018). Edoxaban for the Treatment of Cancer-Associated Venous Thromboembolism. N. Engl. J. Med..

[79] Young A.M., Marshall A., Thirlwall J., Chapman O., Lokare A., Hill C., Hale D., Dunn J.A., Lyman G.H., Hutchinson C. (2018). Comparison of an Oral Factor Xa Inhibitor with Low Molecular Weight Heparin in Patients with Cancer with Venous Thromboembolism: Results of a Randomized Trial (SELECT-D). J. Clin. Oncol..

[80] McBane R.D., Wysokinski W.E., Le-Rademacher J.G., Zemla T., Ashrani A., Tafur A., Perepu U., Anderson D., Gundabolu K., Kuzma C. (2020). Apixaban and Dalteparin in Active Malignancy-Associated Venous Thromboembolism: The ADAM VTE Trial. J. Thromb. Haemost..

[81] Agnelli G., Becattini C., Meyer G., Muñoz A., Huisman M.V., Connors J.M., Cohen A., Bauersachs R., Brenner B., Torbicki A. (2020). Apixaban for the Treatment of Venous Thromboembolism Associated with Cancer. N. Engl. J. Med..

[82] Jegatheswaran J., Hundemer G.L., Massicotte-Azarniouch D., Sood M.M. (2019). Anticoagulation in Patients with Advanced Chronic Kidney Disease: Walking the Fine Line Between Benefit and Harm. Can. J. Cardiol..

[83] Frere C., Font C., Esposito F., Crichi B., Girard P., Janus N. (2022). Incidence, Risk Factors, and Management of Bleeding in Patients Receiving Anticoagulants for the Treatment of Cancer-Associated Thrombosis. Support. Care Cancer.

[84] Salgado M., Brozos-Vázquez E., Campos B., González-Villarroel P., Pérez M.E., Vázquez-Tuñas M.L., Arias D. (2022). Venous Thromboembolism In Cancer Patients: “From Evidence to Care”. Clin. Appl. Thromb. Hemost..

[85] Falanga A., Ay C., Di Nisio M., Gerotziafas G., Langer F., Lecumberri R., Mandala M., Maraveyas A., Pabinger I., Jara-Palomares L. (2023). Venous Thromboembolism in Cancer Patients: ESMO Clinical Practice Guideline ^†^. Ann. Oncol..

[86] Becattini C., Verso M., Muňoz A., Agnelli G. (2020). Updated Meta-Analysis on Prevention of Venous Thromboembolism in Ambulatory Cancer Patients. Haematologica.

[87] Bosch F.T.M., Mulder F.I., Kamphuisen P.W., Middeldorp S., Bossuyt P.M., Büller H.R., van Es N. (2020). Primary Thromboprophylaxis in Ambulatory Cancer Patients with a High Khorana Score: A Systematic Review and Meta-Analysis. Blood Adv..

[88] Cohen A.T., Spiro T.E., Büller H.R., Haskell L., Hu D., Hull R., Mebazaa A., Merli G., Schellong S., Spyropoulos A.C. (2013). Rivaroxaban for Thromboprophylaxis in Acutely Ill Medical Patients. N. Engl. J. Med..

[89] Farge D., Frere C., Connors J.M., Khorana A.A., Kakkar A., Ay C., Muñoz A., Brenner B., Prata P.H., Brilhante D. (2022). 2022 International Clinical Practice Guidelines for the Treatment and Prophylaxis of Venous Thromboembolism in Patients with Cancer, Including Patients with COVID-19. Lancet Oncol.

[90] Hoemme A., Barth H., Haschke M., Krähenbühl S., Strasser F., Lehner C., von Kameke A., Wälti T., Thürlimann B., Früh M. (2019). Prognostic Impact of Polypharmacy and Drug Interactions in Patients with Advanced Cancer. Cancer Chemother. Pharmacol..

[91] Lee J.Y., Oh I.-Y., Lee J.-H., Kim S.-Y., Kwon S.S., Yang H.-J., Kim Y.-K., Bang S.-M. (2020). The Increased Risk of Bleeding Due to Drug-Drug Interactions in Patients Administered Direct Oral Anticoagulants. Thromb. Res..

[92] Steffel J., Collins R., Antz M., Cornu P., Desteghe L., Haeusler K.G., Oldgren J., Reinecke H., Roldan-Schilling V., Rowell N. (2021). 2021 European Heart Rhythm Association Practical Guide on the Use of Non-Vitamin K Antagonist Oral Anticoagulants in Patients with Atrial Fibrillation. Europace.

[93] Peixoto de Miranda É.J.F., Takahashi T., Iwamoto F., Yamashiro S., Samano E., Macedo A.V.S., Ramacciotti E. (2020). Drug-Drug Interactions of 257 Antineoplastic and Supportive Care Agents with 7 Anticoagulants: A Comprehensive Review of Interactions and Mechanisms. Clin. Appl. Thromb. Hemost..

[94] Tsoukalas N., Brito-Dellan N., Font C., Butler T., Rojas-Hernandez C.M., Butler T., Escalante C. (2022). MASCC Hemostasis Study Group Complexity and Clinical Significance of Drug-Drug Interactions (DDIs) in Oncology: Challenging Issues in the Care of Patients Regarding Cancer-Associated Thrombosis (CAT). Support Care Cancer.

[95] Schaefer J.K., Li M., Wu Z., Basu T., Dorsch M.P., Barnes G.D., Carrier M., Griggs J.J., Sood S.L. (2021). Anticoagulant Medication Adherence for Cancer-Associated Thrombosis: A Comparison of LMWH to DOACs. J. Thromb. Haemost..

[96] Khorana A.A., O’Connell C., Agnelli G., Liebman H.A., Lee A.Y.Y. (2012). Subcommittee on Hemostasis and Malignancy of the SSC of the ISTH Incidental Venous Thromboembolism in Oncology Patients. J. Thromb. Haemost..

[97] O’Connell C.L., Boswell W.D., Duddalwar V., Caton A., Mark L.S., Vigen C., Liebman H.A. (2006). Unsuspected Pulmonary Emboli in Cancer Patients: Clinical Correlates and Relevance. J. Clin. Oncol..

[98] Carmona-Bayonas A., Gómez D., Martínez de Castro E., Pérez Segura P., Muñoz Langa J., Jimenez-Fonseca P., Sánchez Cánovas M., Ortega Moran L., García Escobar I., Rupérez Blanco A.B. (2020). A Snapshot of Cancer-Associated Thromboembolic Disease in 2018-2019: First Data from the TESEO Prospective Registry. Eur. J. Intern. Med..

[99] Qdaisat A., Kamal M., Al-Breiki A., Goswami B., Wu C.C., Zhou S., Rice T.W., Alagappan K., Yeung S.-C.J. (2020). Clinical Characteristics, Management, and Outcome of Incidental Pulmonary Embolism in Cancer Patients. Blood Adv..

[100] Gladish G.W., Choe D.H., Marom E.M., Sabloff B.S., Broemeling L.D., Munden R.F. (2006). Incidental Pulmonary Emboli in Oncology Patients: Prevalence, CT Evaluation, and Natural History. Radiology.

[101] Farrell C., Jones M., Girvin F., Ritchie G., Murchison J.T. (2010). Unsuspected Pulmonary Embolism Identified Using Multidetector Computed Tomography in Hospital Outpatients. Clin. Radiol..

[102] Ritchie G., McGurk S., McCreath C., Graham C., Murchison J.T. (2007). Prospective Evaluation of Unsuspected Pulmonary Embolism on Contrast Enhanced Multidetector CT (MDCT) Scanning. Thorax.

[103] Storto M.L., Di Credico A., Guido F., Larici A.R., Bonomo L. (2005). Incidental Detection of Pulmonary Emboli on Routine MDCT of the Chest. AJR Am. J. Roentgenol..

[104] van Es N., Bleker S.M., Di Nisio M. (2014). Cancer-Associated Unsuspected Pulmonary Embolism. Thromb. Res..

[105] Font C., Farrús B., Vidal L., Caralt T.M., Visa L., Mellado B., Tàssies D., Monteagudo J., Reverter J.C., Gascon P. (2011). Incidental versus Symptomatic Venous Thrombosis in Cancer: A Prospective Observational Study of 340 Consecutive Patients. Ann. Oncol..

[106] Sun J.-M., Kim T.S., Lee J., Park Y.H., Ahn J.S., Kim H., Kwon O.J., Lee K.S., Park K., Ahn M.-J. (2010). Unsuspected Pulmonary Emboli in Lung Cancer Patients: The Impact on Survival and the Significance of Anticoagulation Therapy. Lung Cancer.

[107] Shinagare A.B., Guo M., Hatabu H., Krajewski K.M., Andriole K., Van den Abbeele A.D., DiPiro P.J., Nishino M. (2011). Incidence of Pulmonary Embolism in Oncologic Outpatients at a Tertiary Cancer Center. Cancer.

[108] Bach A.G., Schmoll H.-J., Beckel C., Behrmann C., Spielmann R.P., Wienke A., Abbas J., Surov A. (2014). Pulmonary Embolism in Oncologic Patients: Frequency and Embolus Burden of Symptomatic and Unsuspected Events. Acta Radiol..

[109] Connolly G.C., Menapace L., Safadjou S., Francis C.W., Khorana A.A. (2013). Prevalence and Clinical Significance of Incidental and Clinically Suspected Venous Thromboembolism in Lung Cancer Patients. Clin. Lung Cancer.

[110] Kraaijpoel N., Bleker S.M., Meyer G., Mahé I., Muñoz A., Bertoletti L., Bartels-Rutten A., Beyer-Westendorf J., Porreca E., Boulon C. (2019). Treatment and Long-Term Clinical Outcomes of Incidental Pulmonary Embolism in Patients with Cancer: An International Prospective Cohort Study. J. Clin. Oncol..

[111] Di Nisio M., Lee A.Y.Y., Carrier M., Liebman H.A., Khorana A.A. (2015). Subcommittee on Haemostasis and Malignancy Diagnosis and Treatment of Incidental Venous Thromboembolism in Cancer Patients: Guidance from the SSC of the ISTH. J. Thromb. Haemost..

[112] Key N.S., Khorana A.A., Kuderer N.M., Bohlke K., Lee A.Y.Y., Arcelus J.I., Wong S.L., Balaban E.P., Flowers C.R., Francis C.W. (2020). Venous Thromboembolism Prophylaxis and Treatment in Patients with Cancer: ASCO Clinical Practice Guideline Update. J. Clin. Oncol..

[113] Christensen T.D., Vad H., Pedersen S., Hvas A.-M., Wotton R., Naidu B., Larsen T.B. (2014). Venous Thromboembolism in Patients Undergoing Operations for Lung Cancer: A Systematic Review. Ann. Thorac. Surg..

[114] Wang Q., Ding J., Yang R. (2021). The Venous Thromboembolism Prophylaxis in Patients Receiving Thoracic Surgery: A Systematic Review. Asia Pac. J. Clin. Oncol..

[115] Batchelor T.J.P., Rasburn N.J., Abdelnour-Berchtold E., Brunelli A., Cerfolio R.J., Gonzalez M., Ljungqvist O., Petersen R.H., Popescu W.M., Slinger P.D. (2019). Guidelines for Enhanced Recovery after Lung Surgery: Recommendations of the Enhanced Recovery After Surgery (ERAS^®^) Society and the European Society of Thoracic Surgeons (ESTS). Eur. J. Cardiothorac. Surg..

[116] Agzarian J., Hanna W.C., Schneider L., Schieman C., Finley C.J., Peysakhovich Y., Schnurr T., Nguyen-Do D., Linkins L.-A., Douketis J. (2016). Postdischarge Venous Thromboembolic Complications Following Pulmonary Oncologic Resection: An Underdetected Problem. J. Thorac. Cardiovasc. Surg..

[117] Okushi Y., Kusunose K., Okayama Y., Zheng R., Nakai M., Sumita Y., Ise T., Tobiume T., Yamaguchi K., Yagi S. (2021). Acute Hospital Mortality of Venous Thromboembolism in Patients with Cancer From Registry Data. J. Am. Heart Assoc..

[118] Prince R.M., Atenafu E.G., Krzyzanowska M.K. (2015). Hospitalizations During Systemic Therapy for Metastatic Lung Cancer: A Systematic Review of Real World vs Clinical Trial Outcomes. JAMA Oncol..

[119] Rubio-Salvador A.R., Escudero-Vilaplana V., Marcos Rodríguez J.A., Mangues-Bafalluy I., Bernárdez B., García Collado C., Collado-Borrell R., Alvarado Fernández M.D., Chacón López-Muñiz J.I., Yébenes Cortés M. (2021). Cost of Venous Thromboembolic Disease in Patients with Lung Cancer: Costecat Study. Int. J. Env. Environ. Res. Public. Health.

[120] Kirschner M., do Ó Hartmann N., Parmentier S., Hart C., Henze L., Bisping G., Griesshammer M., Langer F., Pabinger-Fasching I., Matzdorff A. (2021). Primary Thromboprophylaxis in Patients with Malignancies: Daily Practice Recommendations by the Hemostasis Working Party of the German Society of Hematology and Medical Oncology (DGHO), the Society of Thrombosis and Hemostasis Research (GTH), and the Austrian Society of Hematology and Oncology (ÖGHO). Cancers.

[121] Macbeth F., Noble S., Evans J., Ahmed S., Cohen D., Hood K., Knoyle D., Linnane S., Longo M., Moore B. (2016). Randomized Phase III Trial of Standard Therapy Plus Low Molecular Weight Heparin in Patients with Lung Cancer: FRAGMATIC Trial. J. Clin. Oncol..

[122] Agnelli G., Gussoni G., Bianchini C., Verso M., Mandalà M., Cavanna L., Barni S., Labianca R., Buzzi F., Scambia G. (2009). Nadroparin for the Prevention of Thromboembolic Events in Ambulatory Patients with Metastatic or Locally Advanced Solid Cancer Receiving Chemotherapy: A Randomised, Placebo-Controlled, Double-Blind Study. Lancet Oncol..

[123] Thein K.Z., Yeung S.-C.J., Oo T.H. (2018). Primary Thromboprophylaxis (PTP) in Ambulatory Patients with Lung Cancer Receiving Chemotherapy: A Systematic Review and Meta-Analysis of Randomized Controlled Trials (RCTs). Asia Pac. J. Clin. Oncol..

[124] Li A., Kuderer N.M., Garcia D.A., Khorana A.A., Wells P.S., Carrier M., Lyman G.H. (2019). Direct Oral Anticoagulant for the Prevention of Thrombosis in Ambulatory Patients with Cancer: A Systematic Review and Meta-Analysis. J. Thromb. Haemost..

[125] Di Nisio M., Porreca E., Candeloro M., De Tursi M., Russi I., Rutjes A.W. (2016). Primary Prophylaxis for Venous Thromboembolism in Ambulatory Cancer Patients Receiving Chemotherapy. Cochrane Database Syst. Rev..

[126] Deitcher S.R., Kessler C.M., Merli G., Rigas J.R., Lyons R.M., Fareed J. (2006). ONCENOX Investigators Secondary Prevention of Venous Thromboembolic Events in Patients with Active Cancer: Enoxaparin Alone versus Initial Enoxaparin Followed by Warfarin for a 180-Day Period. Clin. Appl. Thromb. Hemost..

[127] Lee A.Y.Y., Levine M.N., Baker R.I., Bowden C., Kakkar A.K., Prins M., Rickles F.R., Julian J.A., Haley S., Kovacs M.J. (2003). Low-Molecular-Weight Heparin versus a Coumarin for the Prevention of Recurrent Venous Thromboembolism in Patients with Cancer. N. Engl. J. Med..

[128] Meyer G., Marjanovic Z., Valcke J., Lorcerie B., Gruel Y., Solal-Celigny P., Le Maignan C., Extra J.M., Cottu P., Farge D. (2002). Comparison of Low-Molecular-Weight Heparin and Warfarin for the Secondary Prevention of Venous Thromboembolism in Patients with Cancer: A Randomized Controlled Study. Arch. Intern. Med..

[129] Hull R.D., Pineo G.F., Brant R.F., Mah A.F., Burke N., Dear R., Wong T., Cook R., Solymoss S., Poon M.-C. (2006). Long-Term Low-Molecular-Weight Heparin versus Usual Care in Proximal-Vein Thrombosis Patients with Cancer. Am. J. Med..

[130] Romera A., Cairols M.A., Vila-Coll R., Martí X., Colomé E., Bonell A., Lapiedra O. (2009). A Randomised Open-Label Trial Comparing Long-Term Sub-Cutaneous Low-Molecular-Weight Heparin Compared with Oral-Anticoagulant Therapy in the Treatment of Deep Venous Thrombosis. Eur. J. Vasc. Endovasc. Surg..

[131] Kahale L.A., Hakoum M.B., Tsolakian I.G., Matar C.F., Terrenato I., Sperati F., Barba M., Yosuico V.E., Schünemann H., Akl E.A. (2018). Anticoagulation for the Long-Term Treatment of Venous Thromboembolism in People with Cancer. Cochrane Database Syst. Rev..

[132] Lee A.Y.Y., Kamphuisen P.W., Meyer G., Bauersachs R., Janas M.S., Jarner M.F., Khorana A.A. (2015). CATCH Investigators Tinzaparin vs Warfarin for Treatment of Acute Venous Thromboembolism in Patients with Active Cancer: A Randomized Clinical Trial. JAMA.

[133] Bauersachs R., Berkowitz S.D., Brenner B., Buller H.R., Decousus H., Gallus A.S., Lensing A.W., Misselwitz F., Prins M.H., EINSTEIN Investigators (2010). Oral Rivaroxaban for Symptomatic Venous Thromboembolism. N. Engl. J. Med..

[134] Büller H.R., Prins M.H., Lensin A.W.A., Decousus H., Jacobson B.F., Minar E., Chlumsky J., Verhamme P., Wells P., EINSTEIN–PE Investigators (2012). Oral Rivaroxaban for the Treatment of Symptomatic Pulmonary Embolism. N. Engl. J. Med..

[135] Schulman S., Kakkar A.K., Goldhaber S.Z., Schellong S., Eriksson H., Mismetti P., Christiansen A.V., Friedman J., Le Maulf F., Peter N. (2014). Treatment of Acute Venous Thromboembolism with Dabigatran or Warfarin and Pooled Analysis. Circulation.

[136] Schulman S., Kearon C., Kakkar A.K., Mismetti P., Schellong S., Eriksson H., Baanstra D., Schnee J., Goldhaber S.Z. (2009). RE-COVER Study Group Dabigatran versus Warfarin in the Treatment of Acute Venous Thromboembolism. N. Engl. J. Med..

[137] Agnelli G., Buller H.R., Cohen A., Gallus A.S., Lee T.C., Pak R., Raskob G.E., Weitz J.I., Yamabe T. (2015). Oral Apixaban for the Treatment of Venous Thromboembolism in Cancer Patients: Results from the AMPLIFY Trial. J. Thromb. Haemost..

[138] Büller H.R., Décousus H., Grosso M.A., Mercuri M., Middeldorp S., Prins M.H., Raskob G.E., Schellong S.M., Schwocho L., Hokusai-VTE Investigators (2013). Edoxaban versus Warfarin for the Treatment of Symptomatic Venous Thromboembolism. N. Engl. J. Med..

[139] Carrier M., Cameron C., Delluc A., Castellucci L., Khorana A.A., Lee A.Y.Y. (2014). Efficacy and Safety of Anticoagulant Therapy for the Treatment of Acute Cancer-Associated Thrombosis: A Systematic Review and Meta-Analysis. Thromb. Res..

[140] Schrag D., Uno H., Rosovsky R.P.G., Rutherford C., Sanfilippo K.M., Villano J.L., Drescher M.R., Jayaram N.H., Holmes C.E., Feldman L.E. (2021). The Comparative Effectiveness of Direct Oral Anti-Coagulants and Low Molecular Weight Heparins for Prevention of Recurrent Venous Thromboembolism in Cancer: The CANVAS Pragmatic Randomized Trial. JCO.

[141] Planquette B., Bertoletti L., Charles-Nelson A., Laporte S., Grange C., Mahé I., Pernod G., Elias A., Couturaud F., Falvo N. (2022). Rivaroxaban vs Dalteparin in Cancer-Associated Thromboembolism: A Randomized Trial. Chest.

[142] Frere C., Farge D., Schrag D., Prata P.H., Connors J.M. (2022). Direct Oral Anticoagulant versus Low Molecular Weight Heparin for the Treatment of Cancer-Associated Venous Thromboembolism: 2022 Updated Systematic Review and Meta-Analysis of Randomized Controlled Trials. J. Hematol. Oncol..

[143] O’Connell C., Escalante C.P., Goldhaber S.Z., McBane R., Connors J.M., Raskob G.E. (2021). Treatment of Cancer-Associated Venous Thromboembolism with Low-Molecular-Weight Heparin or Direct Oral Anticoagulants: Patient Selection, Controversies, and Caveats. Oncologist.

[144] Lyman G.H., Carrier M., Ay C., Di Nisio M., Hicks L.K., Khorana A.A., Leavitt A.D., Lee A.Y.Y., Macbeth F., Morgan R.L. (2021). American Society of Hematology 2021 Guidelines for Management of Venous Thromboembolism: Prevention and Treatment in Patients with Cancer. Blood Adv..

[145] Streiff M.B., Holmstrom B., Angelini D., Ashrani A., Elshoury A., Fanikos J., Fertrin K.Y., Fogerty A.E., Gao S., Goldhaber S.Z. (2021). Cancer-Associated Venous Thromboembolic Disease, Version 2.2021, NCCN Clinical Practice Guidelines in Oncology. J. Natl. Compr. Canc Netw..

[146] Becattini C., Di Nisio M., Franco L., Lee A., Agnelli G., Mandalà M. (2021). Treatment of Venous Thromboembolism in Cancer Patients: The Dark Side of the Moon. Cancer Treat. Rev..

[147] Howlett J., Benzenine E., Cottenet J., Foucher P., Fagnoni P., Quantin C. (2020). Could Venous Thromboembolism and Major Bleeding Be Indicators of Lung Cancer Mortality? A Nationwide Database Study. BMC Cancer.

[148] Sigel K., Wisnivesky J.P. (2017). Comorbidity Profiles of Patients with Lung Cancer: A New Approach to Risk Stratification?. Ann. Am. Thorac. Soc..

[149] Hua X., Han S.-H., Wei S.-Z., Wu Y., Sha J., Zhu X.-L. (2019). Clinical Features of Pulmonary Embolism in Patients with Lung Cancer: A Meta-Analysis. PLoS ONE.

[150] Kadlec B., Skrickova J., Merta Z., Dusek L., Jarkovsky J. (2014). The Incidence and Predictors of Thromboembolic Events in Patients with Lung Cancer. Sci. World J..

[151] Zhang Y., Yang Y., Chen W., Liang L., Zhai Z., Guo L., Wang C. (2016). China Venous Thromboembolism VTE Study Group Hypertension Associated with Venous Thromboembolism in Patients with Newly Diagnosed Lung Cancer. Sci. Rep..

[152] Gerotziafas G.T., Taher A., Abdel-Razeq H., AboElnazar E., Spyropoulos A.C., El Shemmari S., Larsen A.K., Elalamy I. (2017). COMPASS–CAT Working Group A Predictive Score for Thrombosis Associated with Breast, Colorectal, Lung, or Ovarian Cancer: The Prospective COMPASS-Cancer-Associated Thrombosis Study. Oncologist.

[153] Launay-Vacher V., Scotté F., Riess H., Ashman N., McFarlane P., Ribic C.C.M., Elalamy I. (2018). Thrombosis and Kidney Disease in Cancer: Comorbidities Defining a Very High Risk Patient: A Position Paper from the Cancer & the Kidney International Network. J. Onco-Nephrol..

[154] Lyon A.R., López-Fernández T., Couch L.S., Asteggiano R., Aznar M.C., Bergler-Klein J., Boriani G., Cardinale D., Cordoba R., Cosyns B. (2022). 2022 ESC Guidelines on Cardio-Oncology Developed in Collaboration with the European Hematology Association (EHA), the European Society for Therapeutic Radiology and Oncology (ESTRO) and the International Cardio-Oncology Society (IC-OS). Eur. Heart J..

[155] Rupa-Matysek J., Lembicz M., Rogowska E.K., Gil L., Komarnicki M., Batura-Gabryel H. (2018). Evaluation of Risk Factors and Assessment Models for Predicting Venous Thromboembolism in Lung Cancer Patients. Med. Oncol..

[156] Bandyopadhyay D., Ball S., Hajra A., Chakraborty S., Dey A.K., Ghosh R.K., Palazzo A.M. (2019). Impact of Atrial Fibrillation in Patients with Lung Cancer: Insights from National Inpatient Sample. Int. J. Cardiol. Heart Vasc..

[157] Farmakis D. (2021). Anticoagulation for Atrial Fibrillation in Active Cancer: What the Cardiologists Think. Eur. J. Prev. Cardiol..

[158] Rolfo C., Meshulami N., Russo A., Krammer F., García-Sastre A., Mack P.C., Gomez J.E., Bhardwaj N., Benyounes A., Sirera R. (2022). Lung Cancer and Severe Acute Respiratory Syndrome Coronavirus 2 Infection: Identifying Important Knowledge Gaps for Investigation. J. Thorac. Oncol..

[159] Conibear J., Nossiter J., Foster C., West D., Cromwell D., Navani N. (2022). The National Lung Cancer Audit: The Impact of COVID-19. Clin. Oncol. (R. Coll. Radiol.).

[160] Kollias A., Kyriakoulis K.G., Lagou S., Kontopantelis E., Stergiou G.S., Syrigos K. (2021). Venous Thromboembolism in COVID-19: A Systematic Review and Meta-Analysis. Vasc. Med..

[161] Kollias A., Kyriakoulis K.G., Stergiou G.S., Syrigos K. (2020). Heterogeneity in Reporting Venous Thromboembolic Phenotypes in COVID-19: Methodological Issues and Clinical Implications. Br. J. Haematol..

[162] Dimakakos E., Gomatou G., Catalano M., Olinic D.-M., Spyropoulos A.C., Falanga A., Maraveyas A., Liew A., Schulman S., Belch J. (2022). Thromboembolic Disease in Patients with Cancer and COVID-19: Risk Factors, Prevention and Practical Thromboprophylaxis Recommendations-State-of-the-Art. Anticancer. Res..

[163] Bernard A., Cottenet J., Bonniaud P., Piroth L., Arveux P., Tubert-Bitter P., Quantin C. (2021). Comparison of Cancer Patients to Non-Cancer Patients among COVID-19 Inpatients at a National Level. Cancers.

[164] Obispo B., Rogado J., Muñoz-Rivas N., Pangua C., Serrano G., Lara M.A. (2022). Infanta Leonor Thrombosis Research Group Prevalence of Thrombosis in Patients with Cancer and SARS-CoV-2 Infection. Med. Clin..

[165] Fenioux C., Allenbach Y., Vozy A., Salem J.-É., Maalouf G., Vieira M., Le Joncour A., Benveniste O., Saadoun D., Frère C. (2021). Differences of characteristics and outcomes between cancer patients and patients with no active cancer hospitalised for a SARS-CoV-2 infection. Bull. Cancer.

[166] Terpos E., Ntanasis-Stathopoulos I., Elalamy I., Kastritis E., Sergentanis T.N., Politou M., Psaltopoulou T., Gerotziafas G., Dimopoulos M.A. (2020). Hematological Findings and Complications of COVID-19. Am. J. Hematol..

[167] Lyman G.H., Kuderer N.M. (2020). Clinical Practice Guidelines for the Treatment and Prevention of Cancer-Associated Thrombosis. Thromb. Res..

[168] ESMO Supportive Care Strategies During the COVID-19 Pandemic. https://www.esmo.org/guidelines/cancer-patient-management-during-the-covid-19-pandemic/supportive-care-in-the-covid-19-era.

[169] Kyriakoulis K.G., Dimakakos E., Kyriakoulis I.G., Catalano M., Spyropoulos A.C., Schulman S., Douketis J., Falanga A., Maraveyas A., Olinic D.-M. (2022). Practical Recommendations for Optimal Thromboprophylaxis in Patients with COVID-19: A Consensus Statement Based on Available Clinical Trials. J. Clin. Med..

[170] Li A., Kuderer N.M., Hsu C.-Y., Shyr Y., Warner J.L., Shah D.P., Kumar V., Shah S., Kulkarni A.A., Fu J. (2021). The CoVID-TE Risk Assessment Model for Venous Thromboembolism in Hospitalized Patients with Cancer and COVID-19. J. Thromb. Haemost..

