# Supplementary material for: Lung Cancer Related Thrombosis (LCART): Focus on Immune Checkpoint Blockade
Source: Cancers (Basel). 2024 Jan 20;16(2):450. doi: 10.3390/cancers16020450 (PMC10814098; doi:10.3390/cancers16020450)
Supplement: Supplementary file 1 [file cancers-16-00450-s001.zip › cancers-2733951-supplementary.pdf]

Treatment	Rate of VTE	Risk of Bleeding	Pharmacokinetic Implications	GI Implications	Haematological Implications
**Chemotherapy + immunotherapy**					
Atezolizumab + carboplatin + etoposide	Low	Yes	-	X (D, C, S, M)	A, T
Atezolizumab + carboplatin+ paclitaxel + bevacizumab	2.6%	Yes	P-gp_s, $CYP3A4_s$	X (D, C, S)	A, T
Durvalumab + carboplatin + etoposide	Low	Yes	-	X (D, C, S, M)	A, T
Nivolumab + ipilimumab + carboplatin + paclitaxel	Low	Yes	P-gp_s, $CYP3A4_s$	X (D, C, S)	A, T
Nivolumab + ipilimumab + carboplatin + pemetrexed	Low	Yes	-	X (D, C, S)	A, T
Pembrolizumab + carboplatin + pemetrexed	Low	Yes	-	X (D, C, S)	A, T
Pembrolizumab + cisplatin + pemetrexed	Low	No	-	X (D, C, S)	A, T
**Chemotherapy**					
Carboplatin + gemcitabine	3%	Yes	-	X (D, S)	A, T
Cyclophosphamide + doxorubicin + vincristine	Low	Yes	P-gp_s, P-gp_{ind}, $CYP3A4_s$	X (D, S)	A, T
Docetaxel	9%	Yes	$CYP3A4_s$	X (D, S)	A, T
Docetaxel + ramucirumab	5%	Yes	$CYP3A4_s$	X (D, S)	A, T
Gemcitabine	Low	No	-	X (D, S)	A, T
Irinotecan	Low	No	$CYP3A4_s$	X (D)	A, T
Lurbinectedin	Low	No	$CYP3A4_s$	X (D)	A, T
Paclitaxel	Low	Yes	P-gp_s, $CYP3A4_s$	X (D, S)	A, T
Pemetrexed	Low	No	-	X (D, S)	A, T
Topotecan	Low	No	P-gp_s	D, M	A, T
Vinorelbine	3.8%	No	P-gp_s, $CYP3A4_s$	X (D, S)	A, T
**Targeted Therapy**					
Afatinib	Low	Yes	-	X (D, M)	Epistaxis
Alectinib	3%	No	$CYP3A4_s$, P-gp_{inh}	X (D)	-
Brigatinib	Low	No	$CYP3A4_s$, $CYP3A4_{ind}$, P-gp_s, P-gp_{inh}	X (D)	-
Ceritinib	Low	No	$CYP3A4_s$, $CYP3A4_{ind}$, P-gp_s	X (D)	A
Crizotinib	1-6%	No	$CYP3A4_s$, P-gp_{inh}	X (D)	A
Dabrafenib + trametinib	6%	Yes	$CYP3A4_s$, $CYP3A4_{ind}$, P-gp_s	X (D)	A, T
Dacomitinib	Low	No	P-gp_s	X (D)	-
Entrectinib	Low	No	$CYP3A4_s$, P-gp_s	X (D)	A
Erlotinib	Low	Yes	$CYP3A4_s$	X (D)	-
Gefitinib	Low	Yes	$CYP3A4_s$	X (D)	-
Larotrectinib	Low	No	$CYP3A4_s$, P-gp_s	X	A
Lorlatinib	Low	No	$CYP3A4_s$, P-gp_{inh}	X (D)	A
Osimertinib	2.1%	No	-	X (D, M)	T
**Immunotherapy**					
Nivolumab	Low	No	-	D, C, S	A, T
Nivolumab+ipilimumab	Low	No		X (D, C, S)	A, T
Pembrolizumab	Low	No	-	X (D, C, S)	A, T

Supplementary Table S1. GI Implications–X (e.g. Nausea/ Vomiting Colitis/ Diarrhea/ Mucositis), D: Diarrhea; A: anaemia; C: Colitis; T: Thrombocytopenia; S: Stomatitis; M: Mucositis Substrate of CYP3A4 (CYP3A4s); Inhibitor of CYP3A4 (CYP3A4inh); Inducer of CYP3A4 (CYP3A4ind); P-gp inhibitor (P-gpinh); P-gp inducer (P-gpInd) P-gp substrate (P-gps). Only Strong Inhibitors and inducers noted. Common or Very Common Adverse events included. The clinical relevance of the Pharmacokinetic Implications is not

known. Risk of VTE was noted as low if no or few (<1%) VTE events were recorded in large clinical trials involving the antineoplastic agents or combinations of antineoplastic agents.
